# On the contribution of internal variability and external forcing factors to the Cooling trend over the Humid Subtropical Indo-Gangetic Plain in India

**DOI:** 10.1038/s41598-018-36311-5

**Published:** 2018-12-21

**Authors:** Reshmita Nath, Yong Luo, Wen Chen, Xuefeng Cui

**Affiliations:** 1 Ministry of Education Key Laboratory for Earth System Modeling, Department of Earth System Science, Tsinghua University, Beijing, 100084 China; 2 Joint Center for Global Change Studies, Beijing, 100875 China; 3 Center for Monsoon System Research, Institute of Atmospheric Physics, Chinese Academy of Sciences, Beijing, 100190 China; 4 School of System Sciences, Beijing Normal University, Beijing, 100875 China

## Abstract

The summer surface air temperature (SAT) in the Humid Subtropical Climate Zone in India, exhibits a significant cooling trend (~*−*3 °C/40 yrs.) in CRU observational data during 1961*–*2000. Here we investigate the contribution of internal and external factors, which are driving this cooling trend. Using the Community Earth System Model-Large Ensemble (CESM-LE), we analyze the historical climate change in presence of internal climate variability. Most of the model ensemble members could reproduce this amplified cooling (<−3 °C) as shown from CRU data. Further analyses reveals that external forcing displays a strong cooling effect over this region, while internal variability displays mixed cooling (in most cases) and warming signals. The signal to noise ratio i.e. the ratio of external forcings and internal climatic variability is less than 1, which indicates that internal climatic variability dominates over the forced response. Furthermore, to quantify the role of different external forcing factors we used the CCSM4 single forcing simulations. The simulation results from CESM-LE and CCSM4 suggest that the cooling trend over the region is primarily due to the combined influence of internal variability (~73%) and partly due to aerosol (~10%) and ozone only forcing, which strongly mask the warming effect of GHG and solar forcing.

## Introduction

The Humid Subtropical Climatic Zone (HSTC) in India, which primarily comprises the Indo-Gangetic Plain (IGP) region of Central India are highly vulnerable to the climatic extremities. Moreover, this region is coming under the direct influence of the Indian summer monsoon (ISM). The HSTC region is the home for ~40% of total population and yields ~50% of total agricultural production of India. Geographically, the HSTC zone is stretching from Punjab in the North to the West Bengal in the East and the probability of drought is higher in this region^1,2^. The IGP is one of the world’s largest plains, which was formed by the rivers like Indus, Ganga, Yamuna, Ghaghara, Gandak and Kosi, in the eastern part of the Indian sub-continent, and they are originated from the Himalayas.

It is known that the Indian Summer Monsoon (ISM) is initiated by unequal surface solar heating of the Indian subcontinent and the Indian Ocean in the pre-monsoon and monsoon season. Therefore, the strength of monsoonal precipitation correlated closely with the land-ocean temperature gradient^3^. In some recent studies, Roxy *et al*.^3^ and Jin and Wang^4^ observed a moderate cooling trend in pre monsoon SAT over the Indian subcontinent during 1950–2002. While since 2002, the pre-monsoon season has reversed in tandem with the monsoonal precipitation revival. This revival of ISM precipitation is driven by a strong warming signature over the Indian subcontinent and slower rates of warming over the Indian Ocean. The continental Indian warming is attributed to a reduction of low cloud due to decreased ocean evaporation in the Arabian Sea, and thus decreased moisture transport to India^4^. Here we observed a strong cooling trend over the HSTC region, however, the factors i.e. the external forcings and internal climatic variability contributing to the trend during the monsoon months (June–August) in the last half of 20^th^ century are not yet explored and need to be analyze in details.

The trajectory of the Earth’s climate is determined by the combined influences of internal climatic variability and anthropogenic climate change^5^. Here the term internal climatic variability attributes the unforced natural variability of the climate system that occurs in absence of external forcing, and includes processes intrinsic to the atmosphere, the ocean, and the coupled ocean-atmosphere system^6–9^. At regional scale the signature of anthropogenic climate change might have masked by the internal variability^6,7,10–12^. In the tropics, due to smaller magnitude of natural variability, the signal of anthropogenic warming appears to emerge significantly, prior to the mid and high latitudes^13–17^.

The latest Intergovernmental Panel on Climate Change reports indicate that, the impacts of anthropogenic forcing factors e.g. greenhouse gas (GHG), aerosol (AS), black carbon (BC), land use (LU), and ozone (OZ) contribute maximum to the warming of atmosphere and ocean. On the other hand, the cooling trend in internal variability masked the warming trend significantly. Therefore, understanding the externally forced climate change and the internal variability is a pressing challenge as evidenced by the considerable range of model sensitivities to the identical set of radiative forcings^5,18^. However, for isolating the relative contribution of anthropogenic climate change and the internally generated climatic variability, it requires a specific climate model with ensembles of simulations and each member is subjected to the identical external forcing.

In the recent decades, the atmospheric carbonaceous aerosols have potential impact on the regional climate, hydrological cycle etc. oversouth Asia^19–21^. Among the major anthropogenic sources, biomass burning, industrial and vehicular emissions have contributed significantly to the total aerosol content of the atmosphere and in particular to the carbonaceous species over northern India and the IGP region^22–24^. The warming potential of these species can influence the atmospheric circulation pattern and the cloud-precipitation efficiency over south and south-east Asia^19,25,26^. The varying nature and strength of emission sources (anthropogenic and natural), transport pathways of chemical constituents and boundary layer dynamics are the dominant factors that contribute to the pronounced seasonal variability^27–30^. In addition, recent reports also linked the weakening of Indian summer monsoon rainfall with large scale deforestation and land use/land cover changes (LULCC)^31^ (Paul *et al*.^31^. Moreover, using high-resolution regional climate model (RegCM4) simulations and prescribed land cover of years 1950 and 2005, it is demonstrated that part of the changes in moderate rainfall events and temperature have been caused by LULCC^32^ (Halder *et al*.^32^ On the other hand, a recent study by Joshi and Rai^33^ reported that the opposite phases of AMO and IPO together modulates the total/moderate rainfall over west central and northeast regions in an asymmetric manner; whereas their warm phase stimulates the heavy rainfall over west central region, while their opposite phases together influences the precipitation extremes over the northeastern region.

Therefore, both the anthropogenic forcings and the internal variability appear to have significant impact on the climatic variability over the HSTC region, under warming scenario. In the present analysis, we investigate the relative contribution of the two factors (external and internal) in driving the cooling trend over HSTC region during the last half of 20^th^ century. It is essential to understand the antecedent conditions that may driving the rapid warming trend after 2000 and revival of ISM over the Indian landmass. The rest of the paper is organized as follows: Section 2 describes the models and experimental design, as well as the observational datasets used to supplement the model simulated trend. Section 3 provides results on the range of climate trends for a historical period of 40 years from each model ensemble, showing the relative contributions from internal variability and external forcing in terms of spatial maps, signal-to-noise ratios, and the likelihood that the trend will have a particular sign (e.g., warming). Section 4 describes the results from CCSM4 single forcing simulations and highlights the relative contribution of the individual forcings. Finally, section 5 concludes, summarizes the main findings and a discussion of the results.

## Data and Methodology

### CESM1-CAM5-BGC-Large Ensemble

Community Earth System Model Large Ensemble (CESM-LE) is designed with an explicit goal of enabling assessment of recent past and near-future climate change (1920–2080) in the presence of internal climate variability. Quiet often the internal climate variability is confused with model error^17^ and at times difficult to disentangle. All CESM-LE simulations use a single CMIP5 coupled climate model: the Community Earth System Model, version 1, with the Community Atmosphere Model, version 5 [CESM1 (CAM5)] with a horizontal resolution, approximately 1° in all model components. The CESM1 (CAM5) consists of coupled atmosphere, ocean, land, and sea ice component models. In addition to land carbon cycle calculations, the CESM-LE simulations also include diagnostic biogeochemistry (BGC) calculations for the ocean ecosystem and the atmospheric carbon dioxide cycle^34–37^. Each CESM-LE ensemble member has a unique climate trajectory because of small round-off level differences in their atmospheric initial conditions. In other words, the CESM-LE ensemble spread results from internally generated climate variability alone. After initial condition memory is lost, within a time span of few weeks in the atmosphere, each member evolves chaotically and is affected by the atmospheric circulation fluctuations, which is the characteristic of a random and stochastic process^7,38^. The CESM-LE experiment, therefore, uniquely enables to assess the relative influence of internal climate variability and forced climate change on the climate system.

Usually, the CESM-LE was started with a multi-century 1850 control simulation with constant preindustrial forcing. The first ensemble member with initial conditions was simulated from a randomly selected date in the 1850 control run: 1 January, year 402 and was integrated forward from 1850 to 2100. Ensemble members 2–30 were all started on 1 January 1920 using slightly different initial conditions and the spread in ensemble members 3–30 was generated by round-off level differences in their initial air temperature fields (order of 10^−14^ K). Except the differences in the initial air temperature field, all the simulations had the same initial conditions. Moreover, all the 35 CESM-LE members share essentially the same ocean initial conditions; the CESM-LE does not sample internal climate variability resulting from differing ocean states.

The historical forcings are applied from 1920 to 2005^39^ and representative concentration pathway 8.5 (RCP8.5) forcing^40,41^ from 2006 to 2080. The CESM-LE simulations used the ozone concentrations calculated by a high-top coupled chemistry–climate model {CESM1 [Whole Atmosphere Community Climate Model (WACCM)]} with specified ozone depleting substances^42^.

### CCSM4 1° 20th Century Single Forcing Simulations

The Community Atmosphere Model (CAM4) is using the Lin–Rood finite volume core, which has much improved representation of deep convection scheme. The horizontal grid used is latitude/longitude with 288 × 200 points, resulting in a uniform resolution of 1.25° × 0.9° in the 1° version, and half the number of grid points in both directions in the 2° version. CAM4 uses 26 layers in the vertical. A freeze-dry modification was implemented to the low cloud parameterization^43^, which has the effect of reducing the amount of wintertime low cloud in the Arctic region. The Froude number coefficient in the gravity wave parameterization was retuned, which improved the CAM4 simulation in the upper atmosphere^44^.

The CCSM4 1850 control runs have the following forcings, which are kept constant during the runs. The incoming solar radiation at the top of atmosphere (TOA) is 1360.9 W m^22^, and the CO_2_ level is set to 284.7 ppm. Aerosol concentrations of sulfate, black and organic carbon, dust, and sea salt are specified from a historical run using the CCSM chemistry component with prescribed emissions^39^, plus a low background level due to volcanic activity. The model was initialized with fields from the end of a previous short coupled run that had slightly different parameter settings. The 1° 1850 control was run for 1300 years, but some very small corrections were made during the run. The CCSM4 model had used gridded emissions of reactive gases and aerosols for use in chemistry model simulations needed by climate models for the Climate Model Intercomparison Program #5 (CMIP5) in support of the Intergovernmental Panel on Climate Change (IPCC) Fifth Assessment report (AR5). The model estimate for the year 2000 inventory, which represents a combination of existing regional and global inventories to capture the best information available at this point; 40 regions and 12 sectors are used to combine the various sources. The region number 26 represents India and it used GFEDv2 inventory seasonality to redistribute total carbon emissions of the region in space and time to improve the carbon emission patterns. The historical reconstruction of each emitted compound, for each region and sector, is then forced to agree with our 2000 estimate, ensuring continuity between past and 2000 emissions. Simulations from two chemistry-climate models are used to test the ability of the emission dataset can capture long-term changes in atmospheric ozone, carbon monoxide, aerosol distributions and their interaction with background atmospheric conditions. The aerosol optical depth and additional aerosol diagnostics are in good agreement with previously published estimates and observations.

The CCSM4 twentieth-century runs begin in January 1850 and end in December 2005. They are forced by time series of solar output, greenhouse gases, aerosols, and volcanic activity. The solar output anomaly time series is described in Lean *et al*.^45^ and is added to the 1360.9 W m^22^ used in the 1850 control run. The CCSM4 volcanic activity is included by a time series of varying aerosol optical depths^46^. The CO_2_ and other greenhouse gases (methane and nitrous oxide) are specified as in the IPCC third assessment report. Atmosphere aerosol burden (sulfate, organic carbon, and sea salt), aerosol deposition (black carbon and dust) onto snow, and nitrogen deposition also vary with time. The burdens and deposition rates were obtained from a twentieth-century run with the CCSM chemistry component active, which is forced with prescribed historical emissions^39^. These concentrations do contain an annual cycle and are linearly interpolated in time from year to year within each month. This leads to a smoothly varying aerosol forcing compared to concentrations found in a fully interactive aerosol model. However, this probably does not affect the long-term trend and impact of these aerosols. The initial conditions for the five members of the CCSM4 twentieth-century ensemble are taken from years 863, 893, 937, 983, and 1031 of the 18 1850 control run, which were chosen to be after the last model correction and to span the range of variability in the North Atlantic meridional overturning circulation^44^. The model experiments, total numbers of single forcing simulations, and the time duration of the individual runs, which are used in this analysis, are listed in Table 1.

**Table 1:** CCSM4 simulations, no. of ensemble members and experiment run period.

Model experiments	No. of ensemble members	Experiment Run Period
Land only forcing	3	1850–2005
GHGs only forcing	3	1850–2005
Aerosols only forcing (EC + BC + SO_4_)	3	1850–2005
Ozone only forcing	2	1850–2005
Solar only forcing	3	1850–2005
Volcanoes only forcing	3	1850–2005
Black Carbon only forcing	1	1850–2005
Sulphate only forcing	1	1850–2005

### Methodology

The projected climate trends in individual model realizations would results from the superposition of external natural forcings, internal climate variability and the anthropogenic external forcing (i.e. GHG increases), i.e.

1 $$Tot.trend=(Ext.Nat.forc.\,+\,Ext.anthro.forc.)+Int.Clim.variab.$$

As we mentioned in the introduction, the individual CESM-LE ensemble members are subjected to an identical radiative forcing and except a small difference in the initial air temperature field, all the simulations have the same initial conditions. After initial condition memory is lost, each member evolves chaotically and the spread is resulting from the internally generated climate variability alone. To illustrate this point, we partition the total trends of the individual model realizations into contributions from the externally forced response and the internal variability^5^. The external factors include both the anthropogenic (GHGs, aerosols etc.) and the natural factors (solar & volcanoes). Therefore, the partitioning can be done by averaging all the ensemble members (represent external forced response), which is constant in all the runs and by subtracting the forced response (i.e. ensemble mean) from the total trend (represents internal climatic variability), respectively. It can be written as,

2 $${(Nat.Variab)}_{1}^{35}={(Tot.Trend)}_{1}^{35}-Ensemble\,Mean\,Trend$$

A quantitative assessment of internal variability and the external forcings can be done using a simple signal-to-noise (SNR) analysis. It can be defined as an absolute value of the forced (ensemble mean) trend divided by the standard deviation of trends across the individual ensemble members. This standard metrics convey useful information about the magnitudes of the forced and internally generated components of future climate change; although they do not convey anything about the spatial coherence of the internal contribution.

3 $$SNR=\frac{Forced}{Natural}=\frac{Mean}{Std}$$

From these large ensembles, we quantified the chances that temperature had increased (or decreased) in the historical periods and also over the coming decades by counting the number of runs with a positive trend divided by the total number of runs for each model. We reiterate that in these model ensembles, the reason why individual runs may show opposite-signed trends at a given location is due to unpredictable, internally generated variability. It can be written as,

4 $$ \% \,warming\,trend=\,\frac{No.of\,runs\,with+ve\,trend}{Total\,no.of\,runs}\times 100$$

Less positive trend (i.e. warming) indicates more negative trend (i.e. cooling).

Relative contribution of the internal climatic variability and the anthropogenic forcings can be calculated as,

5 $$Na{t}_{perc.}=\frac{Std}{[abs(Mean)+Std]}\times 100,Anthr{o}_{perc.}=100-Na{t}_{perc.}$$

The contribution of aerosol, ozone and volcano forcing and natural variability driving the cooling trend can be obtained as,

6 $$Int.va{r}_{perc.}=\frac{Tint}{[Taer+Tozn+Tvol+Tint]}\times 100,$$

and so on for other variables, where, *Tint*, *Taer*, *Tozn*, and *Tvol* represent the trend in internal variability, aerosol only, ozone only and volcano only simulations, respectively.

## Results from CESM1.1

### Overview of SAT trends

**Figure 1:**
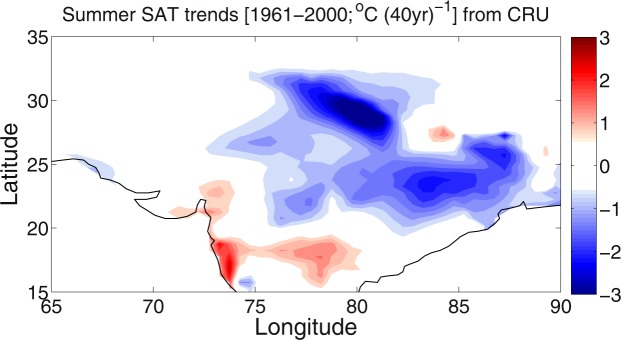
SAT trend from observation. Summer SAT significant (>90%) trends [1961–2000; °C (40 yr)^−1^] from CRU based observation data. The maps in the figure are generated using the MATLAB software (Version: R2012b (8.0.0.783) & http://www.mathworks.com/products/matlab/?s_tid=srchtitle).

**Figure 2:**
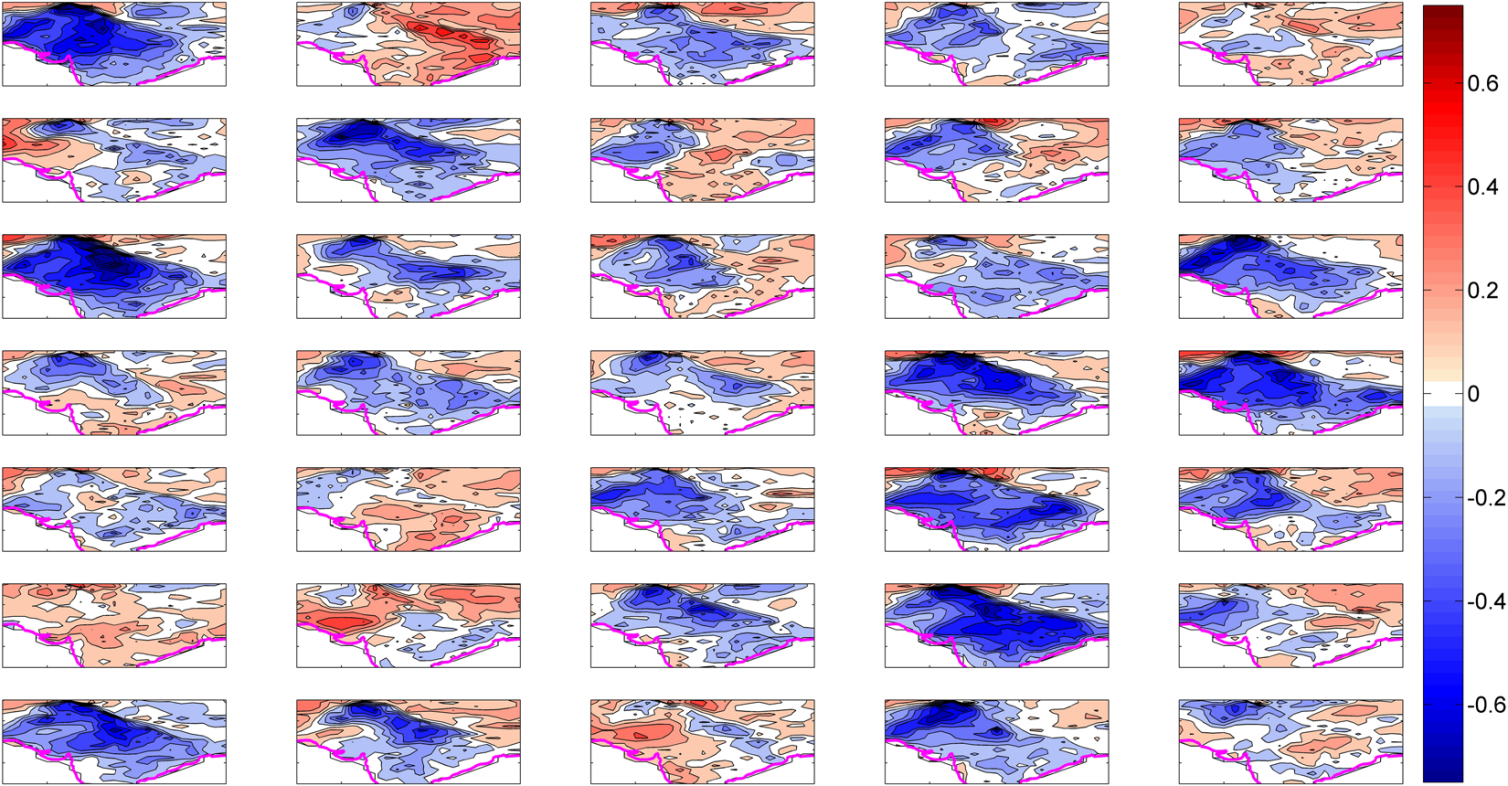
SAT total trend from model simulations. Decadal trends [1961–2000; °C (dec)^−1^] in summer SAT from CESM1 ensemble members (35). The subplot in the top left corner represents member 1 and counting forward towards right. The maps in the figure are generated using the MATLAB software (Version: R2012b (8.0.0.783) & http://www.mathworks.com/products/matlab/?s_tid=srchtitle).

In the beginning, we plotted the summer (JJA) surface air temperature (SAT) trends from the CRU based observation data during 1961–2000 in Fig. 1. A multidecadal cooling trend is predominant over the HSTC zone, particularly in the IGP region. To compare the results with model based simulations, we performed trend analysis over 1961–2000 periods from each of the CESM1 (35 ensemble members) runs, which are presented in Fig. 2. The observation can be seen as a superposition of contribution from the internal climatic variability and the external forcing factors (Equation 1). We compare the temperature trend with NCEP reanalysis data (Supplementary Fig. 1) and the cooling trends are consistent with the CRU based observation data.

In Fig. 2, we show a subset of results to illustrate some key points in the HSTC region. Summer SAT decadal trends over the analysis period (Fig. 2) display considerable diversity across the CESM1 35-member ensemble, despite each simulation being subject to identical radiative forcing. For example, many of the ensemble members exhibit amplified cooling (<−0.75 °C/dec i.e. −3.0 °C/40 years) over the HSTC zone of India (EM 1, 7, 11, 15, 19, 20, 24, 29, 31 and 34), some exhibits mild cooling (EM 3, 4, 6, 12, 14, 16, 17, 18, 21, 23, 25, 28 and 32), while few show weak warming (<0.5 °C/dec i.e. 2.0 °C/40 years) over the region (EM 2, 22, 26, 27 and 33). However, most of the ensemble simulations reflect the cooling trend over the HSTC zone; a pattern resembles with the observation results in Fig. 1.

### Partitioning of total trends into internal and forced components

**Figure 3:**
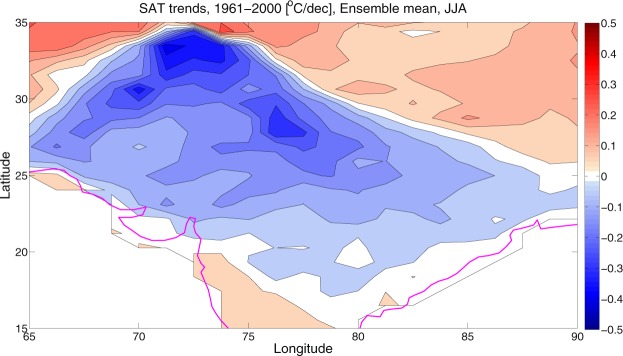
SAT trend due to external forcings. Ensemble mean decadal SAT trends [1961–2000; °C (dec)^−1^]. It represents the externally forced responses. The maps in the figure are generated using the MATLAB software (Version: R2012b (8.0.0.783) & http://www.mathworks.com/products/matlab/?s_tid=srchtitle).

The variety of climate trends in individual model realizations results from the superposition of internal climate variability and the response to external forcing (i.e., GHG increases). To illustrate this point, we partition the total decadal trends into contributions from the externally forced response (obtained by averaging all ensemble members) and the internal variability (obtained by subtracting the forced response from the total trend) (equation 2). Figure 3 represents the spatial pattern and the significant (>95%) decadal trend of the externally forced component (include GHGs, aerosols, ozone, land use and natural external forcings), under the historical scenario. It is generated by averaging the trend of the 35 ensemble members based on CESM1. In Fig. 3, the decadal cooling trends exhibit a continental-scale pattern with maximum amplitudes of approximately <=−3 °C/40 years, which stretches from west to East over most of the HSTC zone. The amplitude of cooling trend maximizes along the IGP region. Several external forcing factors might have responsible for the cooling trend in the HSTC region. In the recent decades, the atmospheric carbonaceous aerosols have potential impact on the regional climate, hydrological cycle etc. over the south Asia^19–21^ and a stronger driver of the atmospheric cooling trend. Among the major anthropogenic sources, biomass burning, industrial and vehicular emissions have contributed significantly to the total aerosol content of the atmosphere and in particular to the carbonaceous species over northern India and the IGP region^22–24^. Additionally, natural external factors like volcano only forcing might have contributed to the cooling trend significantly.

**Figure 4:**
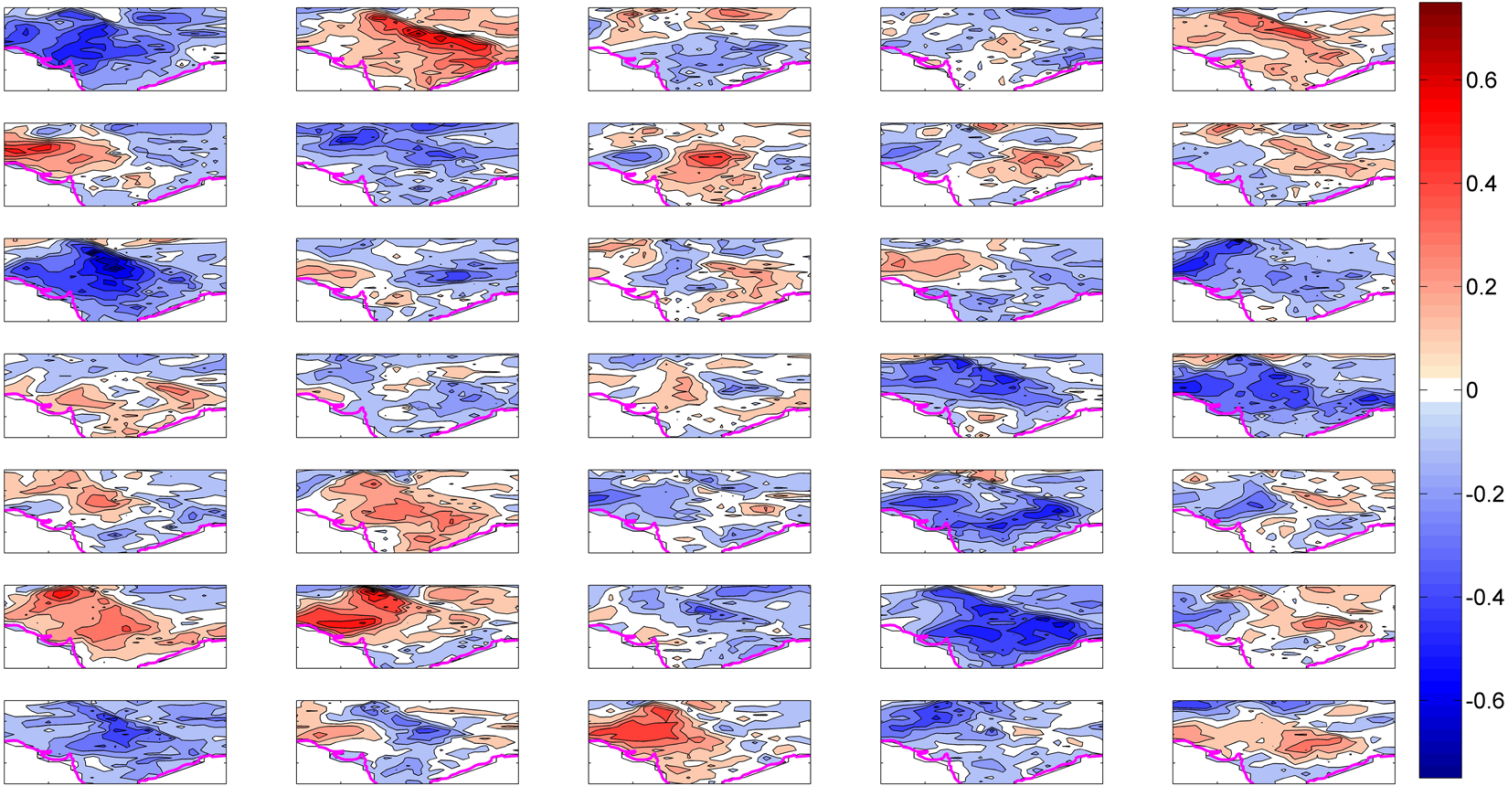
SAT trend due to internal variability. Summer SAT decadal trends after decomposition [1961–2000; °C (dec)^−1^] from the total trends. It actually represents the internal climatic variability among the runs. The subplot in the top left corner represents member 1 and counting forward towards right. The maps in the figure are generated using the MATLAB software (Version: R2012b (8.0.0.783) & http://www.mathworks.com/products/matlab/?s_tid=srchtitle).

The model uncertainties which are referred as internal variability in the CESM ensembles are obtained by subtracting the ensemble mean trend from the total trends. Figure 4, displays the decadal trends resulting from internal climatic variability or model uncertainties under the historical scenario. If we look carefully, in Fig. 4, both the decadal cooling (<=−2 °C/40 years, EM 1, 7, 11, 15, 19, 20, 24, 29, 31 and 34) and warming (>=2 °C/40 years, EM 2, 5, 9, 22,26, 27, 33 and 35) trends appear to dominate the HSTC zone, in particular over the IGP region. Therefore, the internal climatic variability introduces wide range of uncertainty to the climate model simulations. A recent study by Joshi and Rai^33^ provide a deep insight on low-frequency variability and its extremes over India under the combined influence of the Atlantic multidecadal oscillation (AMO) and the interdecadal Pacific oscillation (IPO). The large scale changes in monsoonal circulation might have contributed and impart regional coherence to the internal component of surface climate trends over the HSTC region.

### Quantifying the relative contributions of internal variability and external forcing

**Figure 5:**
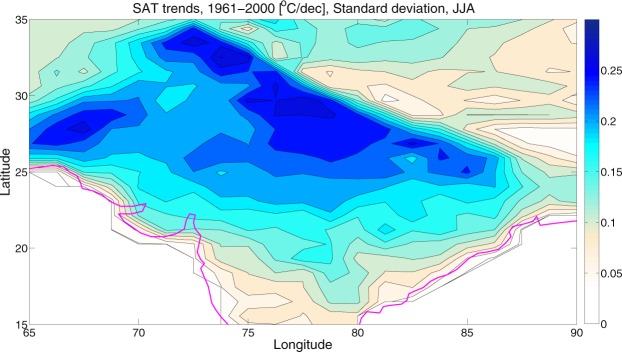
Standard deviation. It represents the same as Fig. 3 but for standard deviation. Higher the standard deviation, higher the contribution of natural variability is. The maps in the figure are generated using the MATLAB software (Version: R2012b (8.0.0.783) & http://www.mathworks.com/products/matlab/?s_tid=srchtitle).

**Figure 6:**
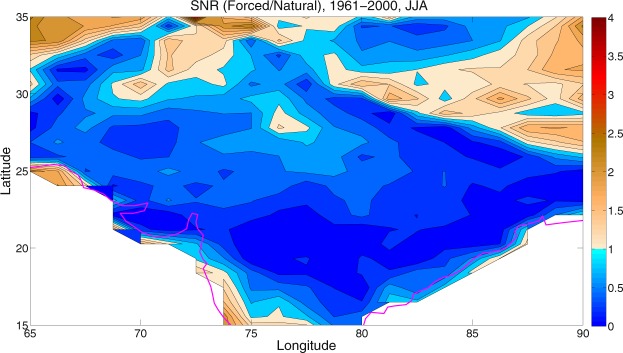
Signal to Noise Ratio. It represents the SNR (forced/natural) during 1961–2000. SNR < 1 indicates relative dominance of natural variability over forced factors. The maps in the figure are generated using the MATLAB software (Version: R2012b (8.0.0.783) & http://www.mathworks.com/products/matlab/?s_tid=srchtitle).

The results shown above give a qualitative impression of the range of patterns and amplitudes of the historical SAT trends over the HSTC zone because of external radiative forcing and internal variability. Here we provide a quantitative assessment using a simple signal-to-noise (SNR) analysis (Equation 3). As mentioned before, the Fig. 3 estimated the forced SAT trends in the CESM1, by averaging over all the ensemble members, under the historical scenario. On the other hand, Fig. 5 shows the standard deviation of the SAT trends (e.g., internal variability) across the ensemble members, during the analysis period. From the standard deviation plot, it is clear that the uncertainty due to natural or internal variability is very strong over the HSTC zone. The signal-to-noise ratio (SNR) between the forced SAT trend and the internal variability is shown in Fig. 6, under the historical scenario. These standard metrics convey useful information about the magnitudes of the forced and internally generated components of late 20^th^ century climate change; although they do not convey anything about the spatial coherence of the internal contribution.

If we look into the spatial pattern of SNR in Fig. 6, the value of SNR is less than 1, throughout the HSTC region. It indicates the relative dominance of the internal climatic variability over the forced response in driving the long term trends in SAT. Therefore, the natural or internal variability appears to masked the warming trend over the HSTC region, to a greater extent.

### Chance of positive trends

**Figure 7:**
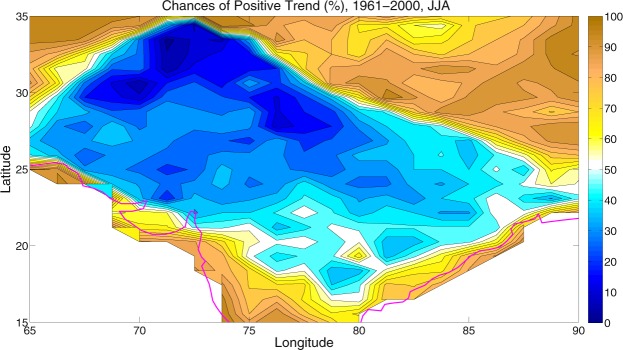
Chances of positive trend. Chances of positive trend or warming (%) during 1961–2000. Note that a low chance implies a high chance of a negative or cooling trend. The maps in the figure are generated using the MATLAB software (Version: R2012b (8.0.0.783) & http://www.mathworks.com/products/matlab/?s_tid=srchtitle).

From these large ensembles, one can quantify the chances that temperature increases (or decreases) and will projected to increase (or decrease) during the historical decades. It can be done by counting the number of runs with a positive trend divided by the total number of runs for each model (Equation 4). It can be reiterated that in these model ensembles, the reason why individual runs may show opposite-signed trends at a given location is due to unpredictable, internally generated climatic variability. Figure 7 shows the results for summer SAT decadal trends for the historical (1961–2000) period. It is noteworthy that a low chance of warming (positive trend) implies a high chance of a negative trend i.e. cooling.

Over the period, the CESM1 model simulations show a higher than 95% chance that summers cools almost over much of the HSTC region, while warming trend dominates elsewhere in the Asian continent (not shown here). In the HSTC region, the relative contribution of anthropogenic warming is partially counteracted by the cooling trend due to internal climatic variability.

## Results from CCSM4

### Single forcing simulation trends

The total climate trends in the individual model realizations would results from the superposition of external natural forcings, internal climate variability and the anthropogenic external forcing (Equation 1). Using the CESM1, we can separate out the external forcings and the internal climatic variability; however, it is extremely difficult to quantify the relative contribution of the individual external forcing factors influencing the total trend.

**Figure 8:**
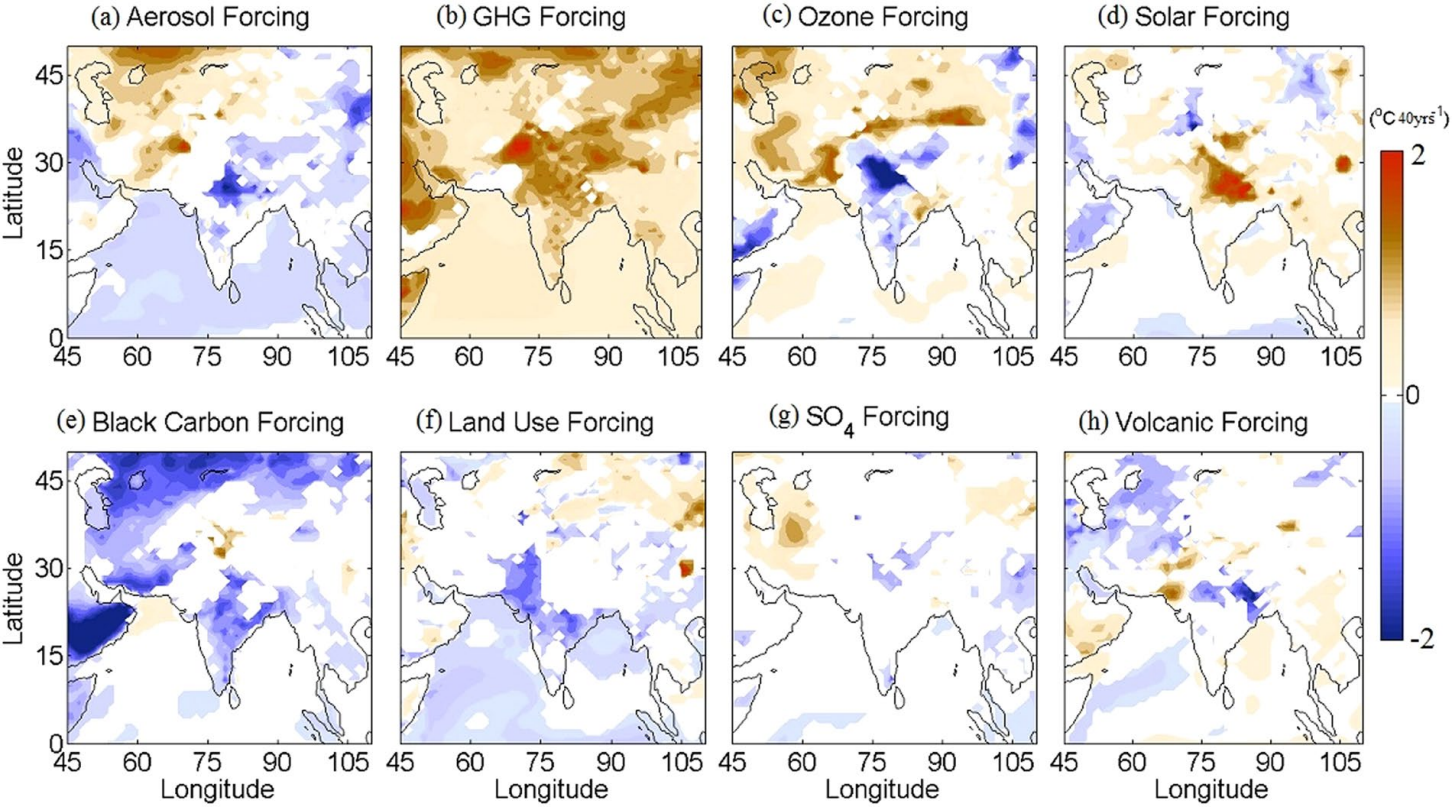
Single forcing trends from CCSM4. Summer SAT trends [1961–2000; °C (40 yr)^−1^] for (**a**) aerosol only (**b**) GHG only (**c**) Ozone only (**d**) Solar only (**e**) Black Carbon only (**f**) Land only and (**h**) Volcanic only forcings from CCSM4. The maps in the figure are generated using the MATLAB software (Version: R2012b (8.0.0.783) & http://www.mathworks.com/products/matlab/?s_tid=srchtitle).

Therefore, to examine the contribution of the individual forcings we have used the CCSM4 single forcing simulations, separately for all forcings, land only, GHG only, aerosol only, ozone only, solar only and volcano only forcings. More details about the model experiments, number of runs, etc. are available in Table 1. The long-term trend in the SAT from the model outputs is analyzed so that the natural and anthropogenic signals in simulated SAT anomaly timeseries (w.r.t. 1971–2000) over India from 1961 to 2000 can be detected during the summer monsoon months (Fig. 8). Results show that the anthropogenic forcing factors such as GHG and aerosols can significantly affect the SAT trend over the HSTC zone (particularly the IGP region). Due to GHG only forcing (Fig. 8b), a warming trend (~2.4 °C/40 yrs) predominates along the IGP region, stretching between 15–35°N and 65–90°E. However, the cooling trend due to rapid increase in aerosol loading (Fig. 8a) and the ozone only forcing (Fig. 8c) can mask the warming effect of GHG to some extent. Despite, the negative contribution of aerosol and ozone, an overall warming trend predominates over this region due to anthropogenic only forcing. The land only forcing (Fig. 8f) has weak contribution to the SAT trend in the HSTC region; however, weak cooling trend in the western and southern peninsular region of India is predominant. In external natural forcing category, the solar only (Fig. 8d) and the volcanic only (Fig. 8h) forcings contributes contrastingly, with strong warming trend (>2.4 °C/40 yrs) and weak cooling trend, over the IGP region, respectively. Therefore, both the anthropogenic only and external natural only forcings have mixed response to the total SAT trend over the Indian region.

**Figure 9:**
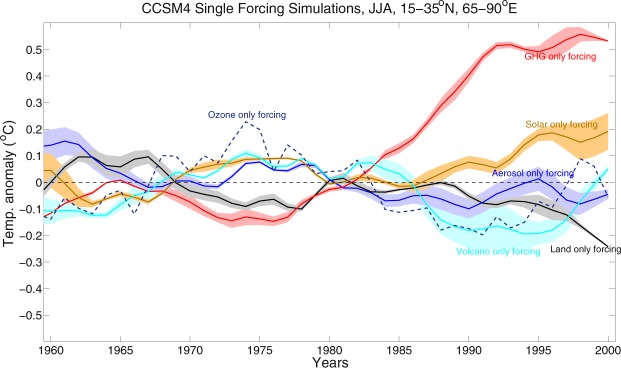
Single forcing timeseries. 11 years smoothed time series of CCSM4 SAT anomalies for GHG only, land only, aerosol only, ozone only, solar only and volcano only over the HSTC zone.

To further investigate the relative contribution of each of the external forcing factors, we plotted the time series of area mean (15–35°N and 65–90°E) SAT anomalies for all the single forcing runs, which are shown in Fig. 9. An 11 years running mean smoothing filter is applied on each of the time series, to remove the inter-annual variability and to highlight the multidecadal variability in the trends. The GHG only and solar only forcings exhibit a stronger warming trends (maximum value reaches 0.55 °C and 0.2 °C, respectively), whereas, the land only, aerosol only, volcano only and ozone only forcing displays cooling trend and the minimum value reaches ~ −0.25 °C, −0.1 °C, −0.2 °C and −0.15 °C, respectively in the recent few decades. Additionally, the internal climatic variability (from CESM1.1 analysis) contribute significantly to the cooling, resulting an overall cooling trend in the HSTC region. We then quantify the relative contribution of individual forcing factors i.e. aerosol, ozone, volcano only forcings and internal variability driving the cooling trend over the HSTC region using Equation 6. The internal variability contributes maximum, which can explain approximately 72% of the temperature variability over that region, whereas, aerosol, ozone and volcano only forcings contribute ~10%, 8.9% and 8%, respectively (Supplementary Fig. 2).

It is worthy to mention that a wide range of uncertainties are involved in climate model simulations in terms of different forcings factors, particularly the aerosol forcing contributes largely to the uncertainties. Lu *et al*.^47^ show that the residential sector contributes the most to overall emission uncertainties in India (60–65% for Black Carbon and 67–74% for Organic Carbon). In CCSM4 single forcings simulations, the uncertainties in aerosol only simulations are approximately 35%, which may result from black carbon and/or sulphate aerosols. Since CCSM4 model have 1 simulation both for sulphate and black carbon only forcing, we cannot quantify their contributions to the uncertainties involved in net aerosols forcings.

## Conclusions and Discussions

We have used the CESM-LE data to assess the climate change in presence of internal climate variability during 1961–2000. The summer (JJA) SAT trend during the 1961–2000 period exhibit an amplified cooling (<*−*3 °C/40 years) in the HSTC region (include IGP). The total trend is partitioned into contributions from the externally forced response and the internal variability. The forced response displays a cooling trend with maximum amplitudes of approximately <=*−*3 °C/40 years during the analysis period in the HSTC zone. The internal climatic variability, also exhibits a strong cooling trend in majority of the ensembles, however, few displays warming trend in the HSTC region. The internal variability, therefore, introduces a wide range of uncertainty in the model simulations. For the analysis period, the SNR is less than 1, throughout the contiguous HSTC region, indicates that the internal climatic variability dominates over the forced response. It is indicative to the fact that, the relative contribution of the natural or internal variability masked the warming trend due to external factors in the HSTC zone, to a greater extent. Moreover, during the analysis period, the CESM1 model simulations show a higher than 95% chance that summers cools almost over much of the HSTC zone.

Next, using the CCSM4 single forcing simulations, we quantify the contribution of individual external forcing factors, separately. The strong warming trend due to the GHG only and Solar only forcings are counteracted by the cooling trend due to the aerosol only, volcano only, land only and ozone only forcings. Additionally, from CESM-LE, the internal climatic variability contribute significantly to the cooling, resulting an overall cooling trend in the HSTC region. Therefore, the strong GHG warming trend is partially counteracted, primarily by the influence of the internal climatic variability (~73%) and partially by certain external forcing factors e.g. aerosol (~10%), volcano, ozone etc. Despite the present study analyzes the historical cooling trend in the HSTC zone, it is essential to study how the near term future trend in SAT will change under strong warming due to anthropogenic forcing factors. Moreover, it is essential to study the variety of natural variabilities and its mechanism which are driving the cooling trend over the region.

## Electronic supplementary material

Supplementary file

## Data Availability

The authors declare to make the data used in this manuscript available anytime on requirement.
