# Supplementary material for: On the contribution of internal variability and external forcing factors to the Cooling trend over the Humid Subtropical Indo-Gangetic Plain in India
Source: Sci Rep. 2018 Dec 21;8:18047. doi: 10.1038/s41598-018-36311-5 (PMC6303293; doi:10.1038/s41598-018-36311-5)
Supplement: Supplementary file 1 — Supplementary file [file 41598_2018_36311_MOESM1_ESM.pdf]

On the contribution of internal variability and external forcing factors to the Cooling trend over the Humid Subtropical Indo-Gangetic Plain in India

Reshmita Nath[1,2], Yong Luo[*1,2], Wen Chen[3], Xuefeng Cui[4]

[1]*Ministry of Education Key Laboratory for Earth System Modeling, Department of Earth System Science, Tsinghua University, Beijing 100084, China*

[2]*Joint Center for Global Change Studies, Beijing 100875, China*

[3]*Center for Monsoon System Research, Institute of Atmospheric Physics, Chinese Academy of Sciences, Beijing 100190, China*

[4]*School of System Sciences, Beijing Normal University, Beijing 100875, China*

[*]Corresponding Author

Yong LUO, Professor
Department of Earth System Science/Institute for Global Change Studies
Tsinghua University
Room S-807, Meng Minwei Science & Technology Building, Tsinghua University
Haidian, Beijing, China 100084
TEL: +86-10-62788891 FAX: +86-10-62797284
E-mail: yongluo@tsinghua.edu.cn

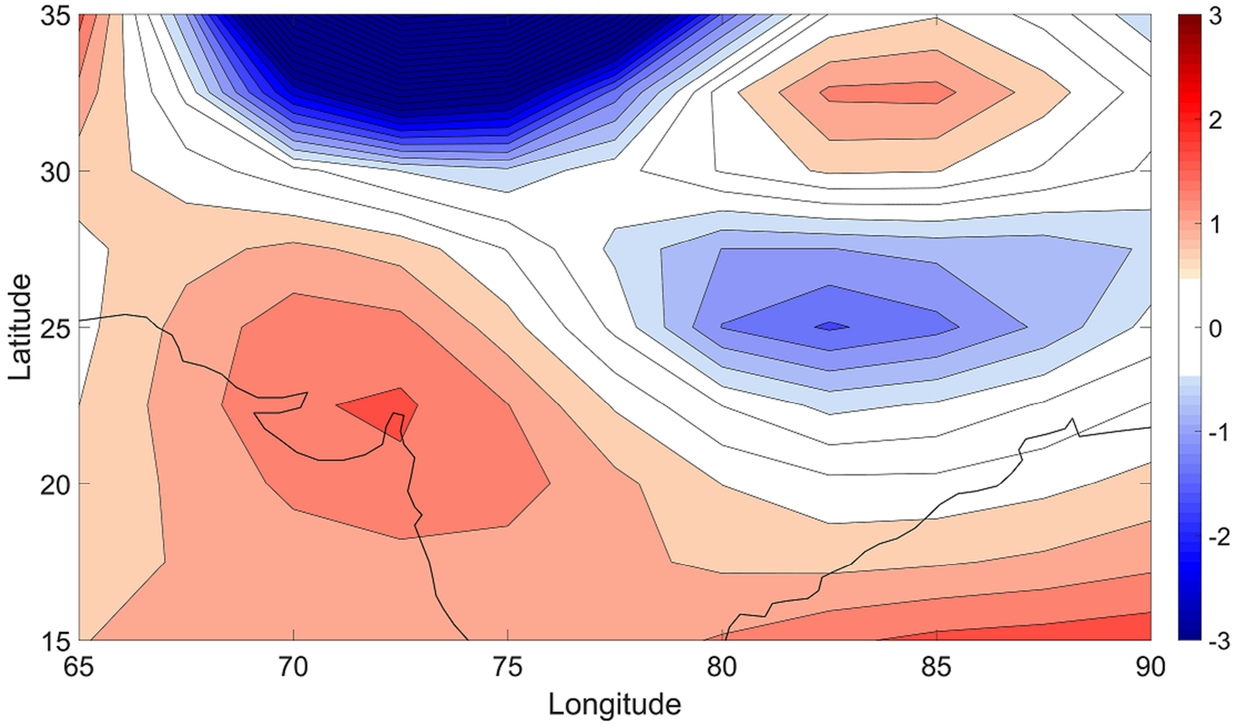

Supplementary Figure 1. SAT trend from NCEP. Summer SAT significant (>90%) trends [1961–2000; °C $(40yr)^{-1}$] from NCEP reanalysis data. The maps in the figure are generated using the MATLAB software (Version: R2012b (8.0.0.783) & URL: http://www.mathworks.com/products/matlab/?s_tid = srchtitle).

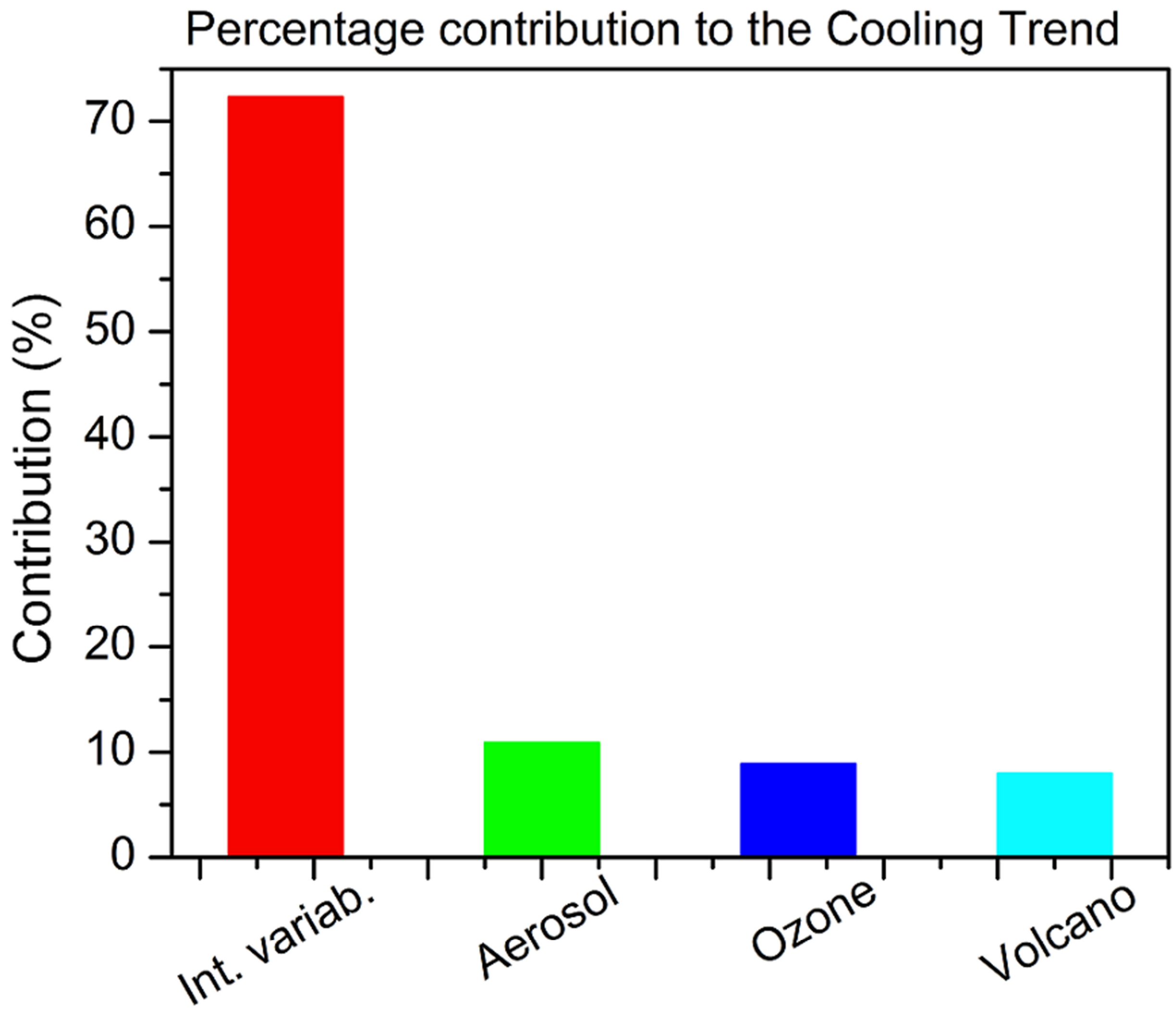

Supplementary Figure 2. Relative contribution (percentage) of individual factors driving the cooling trend over HSTC region.
